# Analysis of Differentially Expressed Transcripts in *Apolygus lucorum* (Meyer-Dür) Exposed to Different Temperature Coefficient Insecticides

**DOI:** 10.3390/ijms21020658

**Published:** 2020-01-19

**Authors:** Jingjie An, Chang Liu, Ya’nan Dou, Zhanlin Gao, Zhihong Dang, Xiu Yan, Wenliang Pan, Yaofa Li

**Affiliations:** 1 Key Laboratory of Integrated Pest Management on Crops in Northern Region of North China, Ministry of Agriculture and Rural Affairs, IPM Center of Hebei Province, Plant Protection Institute, Hebei Academy of Agricultural and Forestry Sciences, Baoding 071000, China; anjingjie147@163.com (J.A.); gaozhanlin@sina.com (Z.G.); dangzhihong@sina.com (Z.D.); pwenliang@163.com (W.P.); 2 College of Plant Protection, Nanjing Agricultural University, Nanjing 210095, China; m18013885867@163.com

**Keywords:** transcriptome, temperature effect, *Apolygus lucorum*, imidacloprid, *β*-cypermethrin, phoxim

## Abstract

The existence of a temperature effect of insecticides frustrated the control of the green plant bug *Apolygus lucorum* (Meyer-Dür). Previous studies mostly focused on the application of insecticides, but the underlying mechanism remains incompletely understood. Here, we report a transcriptome profiling of *A. lucorum* treated by three kinds of temperature coefficient insecticides (TCIs) (positive TCI: imidacloprid, negative TCI: b-cypermethrin and non-effect TCI: phoxim) at 15 °C, 25 °C and 35 °C by using next- and third-generation RNA-Seq methods. A total of 34,739 transcripts were annotated from 277.74 Gb of clean data. There were more up-regulated transcripts than down-regulated transcripts in all three kinds of TCI treatments. Further Venn diagrams indicate the regulatory transcripts and regulatory modes were different at the three temperatures. The responses to imidacloprid involved more detox and stress response transcripts such as cytochrome P450 (CYP450), carboxylesterase (CarE) and catalase (CAT) at 35 °C, which was the case for beta-cypermethrin at 15 °C. UDP-glucuronyltransferase (UGT) and heat shock protein (HSP) transcripts were heavily involved, and thus deserve particular note in the temperature effect of insecticides. This high-confidence transcriptome atlas provides improved gene information for further study on the insecticide temperature effect related physiological and biochemical processes of *A. lucorum*.

## 1. Introduction

Temperature can influence the toxicity (or efficacy) of insecticide [1], which may decrease or increase with temperature increase [2]. Such is the case with different insecticides against different insects or the same insects, and even with different varieties of the same category of insecticides against the same insects [2,3,4,5,6]. The temperature coefficient (TC) shows the relationship between the temperature and toxicity of the insecticide (TOI). It is calculated as the ratio between temperature-dependent activity changes and the temperature range in which the change occurred [7]. The TC is called “positive” if the TOI increases as the temperature increases, “negative” if the TOI decreases as the temperature increases, and “no effect” if the TC is unaffected by temperature increase and therefore has no toxicity difference [8]. Those insecticides are correspondingly called positive TC insecticides (pTCIs), negative TC insecticides (nTCIs), and no effect TC insecticides (neTCIs). Organophosphorus insecticides, carbamate insecticides and neonicotinoid insecticides are generally pTCIs [9,10,11,12,13,14], and pyrethroid insecticides are nTCIs in most cases [2,11,15].

With the widespread planting of transgenic *Bacillus thuringiensis* (Bt) cotton to control the cotton bollworm *Helicoverpa armigera* (Hübner) and subsequent reduction of insecticide use, the mirid bug has gradually increased in population size and gained pest status in northern China [16,17]. The green plant bug *Apolygus lucorum* (Meyer-Dür) is the most destructive, which causes serious economic losses every year [18]. It is widely distributed in China because of its strong environmental adaptability [16]. It can live in a wide temperature range (15–35 °C) and host various crops, vegetables, fruit crops, medicinal plants and pasture plants [19]. Foliar spraying of insecticides is the most common controlling method through the crop’s growing season [20]. Given that TCs vary within insecticide classes and among pest species [3,4], several kinds of insecticides against *A. lucorum* have been tested in our previous studies [13,21,22]. It was found that imidacloprid, hexaflumuron and acetamiprid are pTCIs to this insect, and b-cypermethrin, methomyl and *λ*-cyhalothrin were negatively influenced by temperature. Phoxim, which is slightly influenced by temperature, is categorized as neTCI because its TC value is lower than 2.0 [22].

Factors found to be influencing the temperature effect have been explored in several studies, such as insecticide volatility, stability and insect metabolism [15]. Particularly, the target site of insecticides [23,24], the activities of detoxifying enzymes [24,25], metabolic enzymes [23,24] and heat shock proteins (HSPs) [26,27,28] were addressed. However, one or several components could not provide a systematic understanding of the whole mechanism and pathways associated. Recent decades have seen increasingly rapid advances in the field of high throughput sequencing, which can comprehensively elucidate the types and number of genes, and reveal the physiological and biochemical processes in organisms under certain conditions [29]. Next-generation transcriptome sequencing was also used on *A. lucorum* [30,31,32]. However, there have been no transcriptomic studies which dealt with temperature effect of insecticides and the next-generation sequencing approach prevented the assembly of full-length transcripts from the short reads to non-model organisms without reference genome in nature. Single-molecule real-time (SMRT) sequencing carried out in PACBIO RS (Pacific Biosciences of California, Inc., CA, USA) recently overcame the limitations of short-read sequences and took the advantages of the higher accuracy to identify alternative isoforms and analyze the alternative splicing (AS) events that help better understand the physiological and biochemical processes [33,34].

This study set out to investigate the molecular mechanisms of temperature effect on the toxicity of insecticides on *A. lucorum*. We performed transcriptomic analysis of nymphs treated with three kinds of TCIs (imidacloprid, beta-cypermethrin and phoxim) at different temperatures (15, 25 and 35 °C). The full-length transcriptome was obtained by the SMRT sequencing method to the whole mixture samples. This combination of second- and third- generation sequencing will provide new insights into gene sequence characteristics and make contributions to our increased understanding of the insecticide temperature effect related physiological and biochemical processes of *A. lucorum*.

## 2. Results

### 2.1. De Novo Assembly, Functional Annotation and Transcripts Analysis

We performed RNA-seq to quantify the expression of transcripts of *A. lucorum* after the sub-lethal dose treatment of imidacloprid (IM), beta-cypermethrin (BCP), phoxim (PHX) at 15 °C, 25 °C and 35 °C respectively (these samples were named as IM15, IM25, IM35, BCP15, BCP25, BCP35, PHX15, PHX25 and PHX35). A total of 277.74 Gb clean data were obtained from the short-read RNA sequencing of 36 samples, and the Q30 reached 90.52% (Table S1). The mixed 36 RNA samples were collected for library preparation of SMRT sequencing. Three size fractions (1–2 kb, 2–3 kb, 3–6 kb) of sample totally generated 1,202,336 polymerase reads and 19.65 Gb of clean reads. From them, 507,176 reads of insert (ROIs) were successfully obtained, and 243,126 full-length non-chimeric (FLNC) sequences were identified (Table 1). Based on the iterative clustering for error correction (ICE) results, 65,741 consensus isoforms were obtained. We obtained 45,469 transcripts after removing redundant sequences from 48,558 high-quality transcripts.

**Figure 1:**
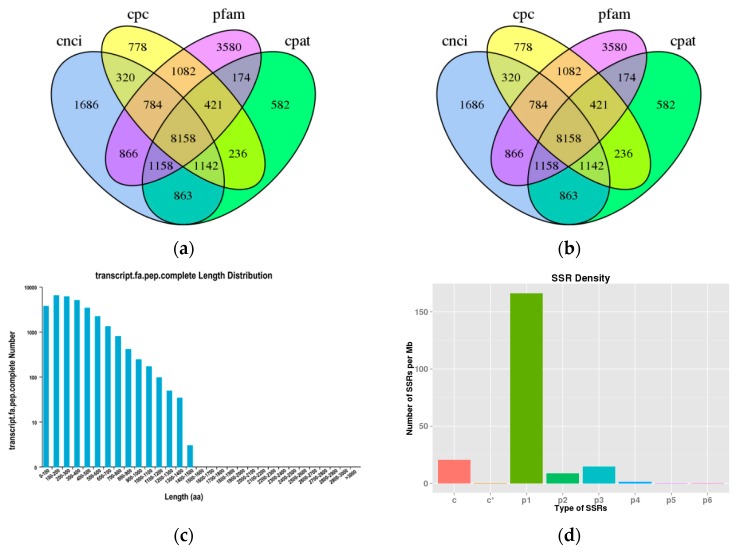
Functional annotation and structural analysis of the full-length transcriptome. (**a**) Functional annotation of non-redundant (NR), gene ontology (GO), clusters of orthologous groups (COG), euKaryotic ortholog groups (KOG), Kyoto Encyclopedia of Genes and Genomes (KEGG), Pfam, non-supervised orthologous groups (eggNOG) and SwissProt database results. (**b**) Venn diagram of the putative lncRNAs predicted using coding-non-coding index (CNCI), coding potential calculator (CPC), coding potential assessment tool (CPAT) and protein family (Pfam) database. (**c**) Coding sequence (CDS) length distribution predicted using TransDecoder. (**d**) Distribution of simple sequence repeats (SSRs) type. p1: Mono-nucleotide; p2: di-nucleotide; p3: tri-nucleotide; p4: tetra-nucleotid; p5: penta-nucleotide; p6: hexa-nucleotide; c: compound SSR.

Based on non-redundant (NR), Swiss-Prot, gene ontology (GO), clusters of orthologous groups (COG), euKaryotic ortholog groups (KOG), protein family (Pfam), evolutionary genealogy of genes: non-supervised orthologous groups (eggNOG), and Kyoto Encyclopedia of Genes and Genomes (KEGG), a total of 17,067 transcripts were annotated in GO database, 17,402 in KEGG, 24,916 in KOG, 26,771 in Pfam, 22,813 in Swiss-Prot, 12,445 in COG, 32,825 in eggNOG and 34,213 in NR (Figure 1a). In addition, 34,739 transcripts were annotated in all of the eight databases (Table S2).

We used four computational approaches to identify lncRNAs from the 48,558 PacBioIso-Seq isoforms. By filtering and excluding transcripts with an open reading frame (ORF) >300 bp, we finally obtained 8158 lncRNAs (Figure 1b).

According to TransDecoder, 36,968 ORFs, including 30,019 complete ORFs, were identified. The distribution of the coding sequence lengths of complete ORFs is shown in Figure 1c.

A total of 30,752 simple sequence repeats (SSRs) were detected and only transcripts >500 bp were subjected to the MISA for analysis. The number of mono-nucleotides, di-nucleotides, tri-nucleotides, tetra-nucleotids, penta-nucleotides, hexa-nucleotides and compound SSRs was 20,977, 1112, 1849, 173, 11, eight and 2604, respectively (Figure 1d).

**Figure 2:**
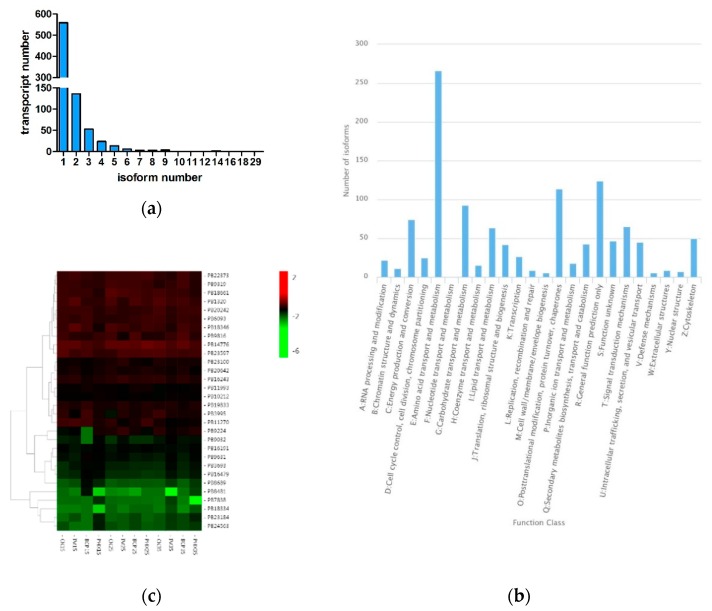
Alternative splicing analysis of the full-length transcriptome. (**a**) Distribution of the transcripts that have one or more alternative isoforms. (**b**) KOG annotation of the transcripts with one or more alternative isoforms. (**c**) Expression heatmap of the transcript with the most isoforms.

We detected 1397 AS events. The distribution of the transcripts that have one or more alternative isoforms is shown in Figure 2a. The transcript with the most isoforms was PB7838 and was annotated as trypsin precursor (Table S3). KOG annotation showed that most transcripts function in amino acid transport and metabolism, followed by general function prediction only and post-transcriptional modification, protein turnover and chaperones (Figure 2b). The expression heatmap of the transcript with the most isoforms showed that PB22373 was the highest expressed isoform and some isoforms were differentially expressed in different samples (Figure 2c).

### 2.2. Identification of Differentially Expressed Transcripts and qRT-PCR Validation

**Figure 3:**
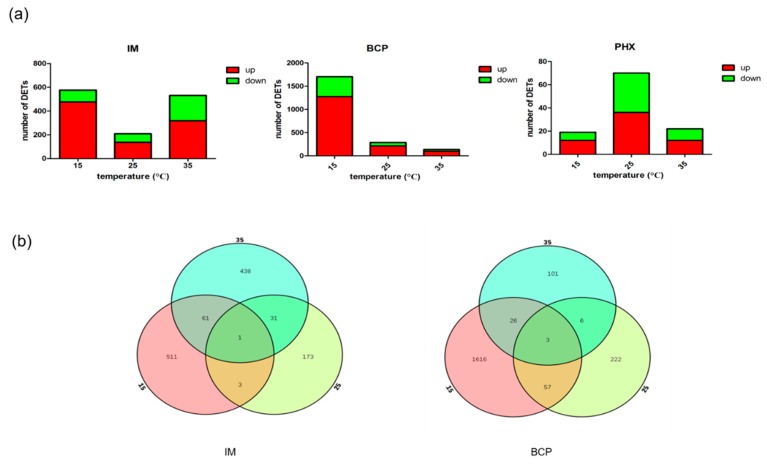
Statistics of differentially expressed transcripts by three insecticides treatments at different temperatures. (**a**) Number of individual transcripts significantly up- or down-regulated with different treatments. (**b**) Venn diagrams illustrating the number of transcripts by insecticides treatment at different temperatures.

The expression levels of the transcripts from the IM, BCP and PHX treated samples were compared to that from the control samples of the same temperature. The differentially expressed transcripts (DETs) were identified using standard for the false discovery rate <0.01 and fold change >2. In the IM treatment group, there were 576, 208 and 531 DETs at 15, 25 and 35 °C, respectively. There were more up-regulated DETs than down-regulated DETs whereas the down-regulated DETs were more at 35 °C than that of other temperatures. For the BCP treatment group, the DETs declined obviously with the increase of temperature. Similar to IM treatment, there were more up-regulated DETs than that of down-regulated in BCP treatment group. The DETs in PHX treatment were far less than IM and BCP treatments, with only 19, 70 and 22 DETs at 15, 25 and 35 °C, respectively (Figure 3a).

To determine whether the DETs are the same transcripts that work at different temperatures, we plotted Venn diagrams of the treatments from 15 °C to 35 °C. Since there were no common DETs in PHX, we did not plot Venn diagram of this group. There were only one and three co-regulated DETs in the IM and BCP respectively, indicating that the regulatory transcripts and regulatory modes were different at the three temperatures. There were 62 common DETs of IM treatments at 15 °C and 35 °C and 60 common DETs of BCP at 15 °C and 25 °C (Figure 3b).

As there were more up-regulated DETs than down-regulated DETs in most cases of this research, we performed GO enrichment on the up-regulated DETs. It can be seen in Table 2 that exopeptidase activity was enriched in IM15, IM25 and BCP15. Furthermore, 1,4-alpha-glucan branching enzyme activity was the most enriched GO term in IM35. Energy metabolism related GO terms, such as AMP binding, NAD binding, and isocitrate dehydrogenase (NADP^+^) activity were enriched in IM35, BCP35 and PHX35, respectively (Table 2).

**Figure 4:**
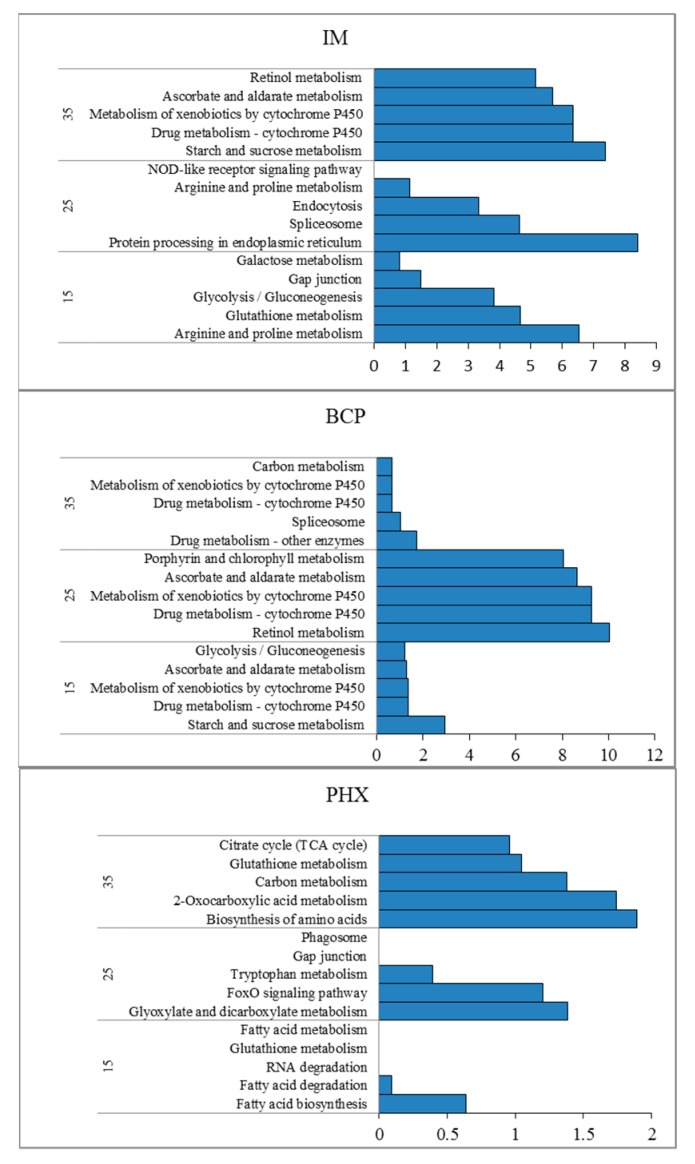
KEGG pathway enrichment analysis of the top five pathways in three treatments. IM is the abbreviation of imidacloprid, BCP is beta-cypermethrin, and PHX is phoxim. Enrichment scores are shown as −log10(*q*).

To further understand the functions of those DETs involved in insecticides and temperature responses, the KEGG pathway enrichment analysis was carried out. For IM15, the enriched pathways included arginine and proline metabolism, glutathione metabolism, glycolysis/gluconeogenesis, gap junction, and galactose metabolism (Figure 4). Further analysis indicated that expression of GSH pathway downstream transcripts was enhanced and the synthesis of energy supply was inhibited. At the same time, the synthesis of ornithine was inhibited and its metabolism was strengthened in proline and arginine metabolic pathways (Figure S1). At 25 °C, protein processing in endoplasmic reticulum, spliceosome, and endocytosis pathways were significantly enriched (*q* < 0.01). Among them, the pathway of protein processing in endoplasmic reticulum mainly involved HSP70, HSP90 and other heat excitatory protein transcripts. It is significantly different from that at 15 °C. At 35 °C, the pathways of starch and sucrose metabolism, drug metaboly-cytochrome P450, metabolism of xenobiotics by cytochrome P450 (CYP450), ascorbate and aldarate metabolism and retinol metabolism were significantly enriched. Most of the transcripts are UDP-glucuronyltransferase (UGT) coding transcripts, indicating that UGT plays an important role at this temperature. For BCP treatment, drug metabolism-cytochrome P450, metabolism of xenobiotics by CYP450 and retinol metabolism were all enriched at the three temperatures. Among them, the enrichment degree of these pathways was significantly higher at 25 °C than that of the other two temperatures. Further analysis showed that UGT coding transcripts were heavily involved in these pathways (Table S4). For PHX, although there were less DET at the three temperatures, glyoxylate and dicarboxylate metabolism, biosynthesis of amino acids, 2-oxocarboxylic acid metabolism and carbon metabolism pathways were significantly enriched (*q* < 0.05).

### 2.3. Pattern Analysis of Temperature Effect Related Transcripts

**Figure 5:**
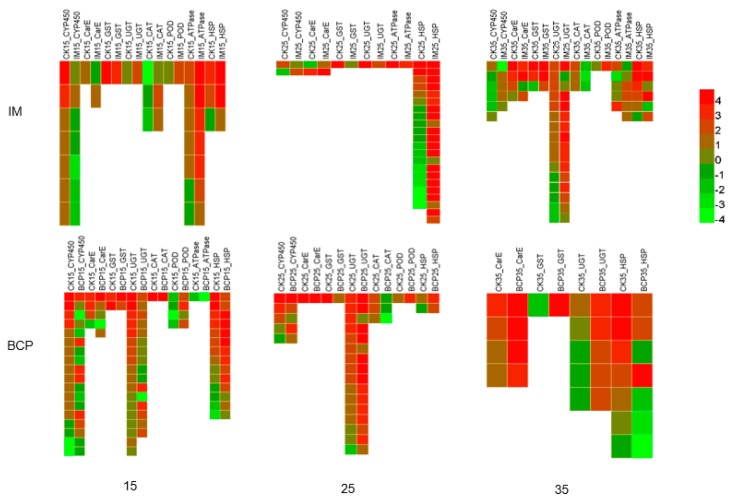
The pattern of temperature effect related differentially expressed transcripts (DETs). These related genes included detoxification enzyme cytochrome P450 (CYP450), glutathione S-transferase (GST), carboxylesterase (CarE), UDP-glucuronyltransferase (UGT), antioxidant enzyme superoxide dismutase (SOD), catalase (CAT), and peroxidase (POD) and adenosine triphosphatase (ATPase) heat shock proteins (HSPs). Compared to the blank control, the enzymes labeled in the red box are associated with up-regulation. The green one means that it was associated with down-regulation.

The patterns of the temperature effect related transcripts were analyzed in both IM and BCP treatments as they have more DETs at the three temperatures. DETs coding detoxification enzymes CYP450, carboxylesterase (CarE), glutathione S-transferase (GST) and UGT, antioxidant enzymes, superoxide dismutase (SOD), catalase (CAT), and peroxidase (POD), adenosine triphosphatase (ATPase) and HSPs were selected for expression mode analysis (Figure 5). It was found that after IM treatment, all detoxification enzyme transcripts were down-regulated except UGT, which was up-regulated at 15 °C, while antioxidant enzyme transcripts ATPase and HSP transcripts were up-regulated. At 25 °C, HSPs, including HSP70, HSP90 and HSP21.4 were up-regulated. Detoxification enzyme transcripts showed no consistent regular pattern, and antioxidant enzyme transcripts were not involved. At 35 °C, various types of enzyme transcripts were widely involved. UGT, as the main metabolic enzyme, was mostly up-regulated and some were down-regulated. Detoxification enzyme transcripts CYP450 and CarE were down-regulated and GST was up-regulated.

In the BCP treatments, most UGT transcripts were down-regulated at 15 °C and up-regulated at 25 °C and 35 °C. CYP450 transcripts with differential expression decreased with the increase of temperature. There was no different expression of antioxidant enzyme transcripts at 35 °C. CarE and GST transcripts were up-regulated at 35 °C. CAT transcripts were up-regulated at the low temperature and down-regulated at the moderate temperature, whereas POD transcripts were always up-regulated.

### 2.4. Validation of RNA-seq by Quantitative Reverse-Transcription PCR

**Figure 6:**
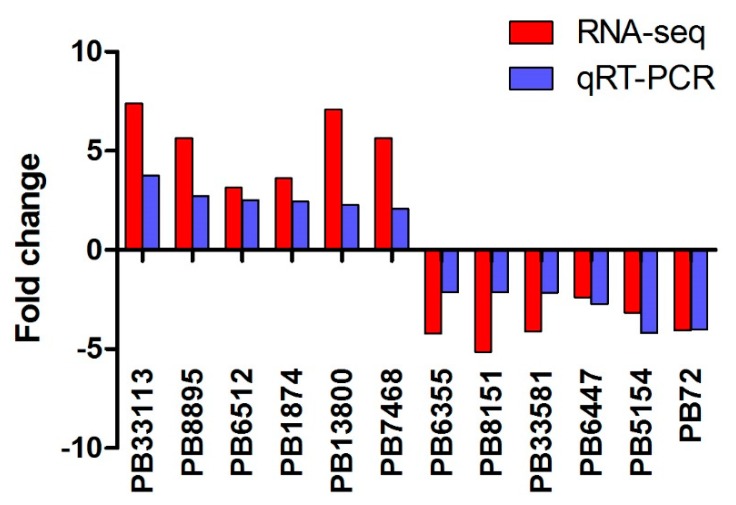
Validation of RNA-seq by quantitative reverse-transcription PCR. Twelve transcripts with different expression levels were identified using qRT-PCR. The y-axis indicates the relative expression levels (Fold change) based on DET analysis and qRT-PCR. Three independent replications were set.

Twelve differentially expressed transcripts were selected to validate the reliability of the RNA-seq data by quantitative reverse-transcription PCR (qRT-PCR). The results show that most of these genes exhibit similar expression patterns between RNA-seq data and qRT-PCR results (Figure 6).

## 3. Discussion

Transcriptome analysis provides a comprehensive understanding of molecular mechanisms involved in specific biological processes. Development of de novo assembly expands the applicability of second generation RNA sequencing especially to non-model organisms without a proper reference genome, such as *A. lucorum* [32]. However, the next-generation RNA sequencing transcriptome analyses in species without reference genome sequences often encounter assembly problems [35]. Therefore, a high-confidence transcriptome atlas is needed to help us better understand and control this important pest species. In the present study, we determined the gene expression of *A. lucorum* treated with IM, BCP, PHX at 15 °C, 25 °C and 35 °C by using second- and third-generation RNA-Seq method. After extensive sequencing and bioinformatic analysis, a total of 34,739 transcripts were annotated from 277.74 Gb clean data. Compared with the assembled transcripts obtained from next-generation projects of *A. lucorum* [30,31,32], the number of long-length and average length of transcripts were greatly improved in our dataset (Table 1), indicating the advantages of the SMRT sequencing.

The fact that full-length transcriptome sequencing generated mRNA molecules with intact 5′- and 3′-UTR sequences allowed us to analyze gene sequence characteristics, which are deficient in previous reports of *A. lucorum* transcriptome. In the present study, we identified 8158 lncRNAs, 30,752 SSRs and 1397 AS events. It is interesting to note that among the 1397 AS events, seven out of the 10 transcripts with the most isoforms were annotated as trypsin. A possible explanation for this might be that *A. lucorum* feeds on a wide range of plants that emit complex and species specific compounds [16], which needs more isoforms to adapt to the digestion of these compounds. Furthermore, it is also reported to contribute to insecticide detoxification [36].

The effect of temperature on the toxicity of insecticides is a complex physiological and biological process and is not a fixed pattern. It is generally believed that temperature increase can enhance penetration of insecticides, and result in an increase in TOI [37], whereas it was not found in nTCIs [38]. Wang et al. [39] found that allethrin, belonging to nTCI, blocked the action potential of squid giant axons more strongly at 8 °C than at 23 °C, and therefore accounted for the negative TC of nerve blocking action of allethrin. Different kinds of pyrethroids, although all interact with the sodium channels, could show different temperature effects [40]. However, very little was found in the literature on the underlying mechanisms, especially the molecular mechanisms. 

In this study, there were more up-regulated DETs than down-regulated DETs in all three kinds of TCI treatments, suggesting that up-regulation was the main response of most transcripts. Interestingly, DETs were less in IM25 than that of IM15 and IM35 (Figure 5), which was not in agreement with the tendency of imidacloprid toxicity. Venn diagrams indicated the regulatory genes and regulatory modes were different at the three temperatures (Figure 3). GO enrichment showed that terms related to energy metabolism were enriched at 35 °C in all insecticides (Table 2), implicating that the high temperature activated energy metabolism regardless the application of insecticides. Further KEGG enrichment showed that detoxification pathways such as drug metabolism-cytochrome P450 and metabolism of xenobiotics by cytochrome P450 were enriched in IM35, BCP15 and BCP25. Surprisingly, UGTs instead of CPY450s were differentially expressed in these pathways. These results are in accordance with previous studies indicating that insect UGT plays vital a role in the detoxification of substrates such as plant allelochemicals and insecticides [41,42].

The detoxification and stress responses of xenobiotics generally include three major and interrelated pathways, that is, oxidation-reduction, conjugation, and hydrolysis [43]. During these processes, mixed function oxidase (CYP450), glutathione S-transferase (GSTs), and esterase are considered to be the most important detox enzymes [44]. In addition, oxidation and reduction enzymes, ATPase and HSPs are frequently involved [43,45]. Consistent with the literature, genes coding these enzymes were also found to be differentially expressed. However, the number and expression pattern varied. For instance, CYP450s were not differentially expressed in BCP35. Most UGT transcripts were down-regulated in BCP treatments at 15 C, but up-regulated in IM treatments. CarE were down-regulated in IM35, whereas significantly up-regulated in BCP35. It is interesting to note that HSPs participated in almost all the treatment but were heavily involved in IM25. HSPs are a family of proteins that can help to protect organisms from induced environmentally cellular damage induced by thermal stress and insecticides exposure [45,46,47]. Up-regulation of HSPs in this study corroborates these earlier findings, with only an exception in BCP35, where most HSPs were down-regulated. The reasons need to be further investigated. Notably, the expression pattern in PHX treatment group was not analyzed in detail, because the DETs were less and little detox and stress response genes were found, which is contrary to the findings of Gu et al. [47]. This inconsistency may be due to the dose used in this study.

In summary, our data provides a high-confidence transcriptome atlas by conducting next- and third-generation sequencing. The results here facilitate our understanding of the molecular mechanisms of temperature coefficient of insecticides. For pTCIs such as IM, the responses involved more detox and stress response genes at 35 °C, which was the case for BCP at 15 °C. At different temperatures, insects regulate these genes precisely according to the characteristics of xenobiotics, thus showing different regulatory modes. The function of UGT and HSP transcripts may be of particular note in the temperature effect of insecticides. To clearly clarify the mechanism of temperature effect of insecticide, further studies need to be undertaken: (1) the results of this study still need to be further verified by RNAi technology or gene silencing technology; (2) as the transcriptomic-analysis results are at a certain time-point in the insect physiological and biochemical process, the changes at different processing times need to be further studied to make them become a dynamic change system.

## 4. Material and Methods

### 4.1. Insects Rearing and RNA Sample Preparation

*A. lucorum* adults were collected from Xian County (38.289° N, 115.550° E), Hebei Province in 2009, and were continuously cultured in the laboratory without exposure to insecticides. All larvae, adults, and eggs were held at 25 ± 1 °C, 60 ± 5%RH and a photoperiod of 16:8 (L:D) h. No less than 100 tested third-instar larvae in one treatment were put in containers, fed with asparagus bean pods dipped of IM, BCP or PHX at (10% LC_50_) for 48 h at 15 °C, 25 °C and 35 °C respectively. The mirids were frozen by liquid nitrogen and stored at −80 °C for further experiment. Thirty-six total RNA samples (four different treatments with three repetitions) were isolated using the RNeasy Plus Mini Kit (Qiagen, Valencia, CA, USA). The total RNA was quantified and the quality was assessed using an Agilent 2100 system (Agilent Technologies, Palo Alto, CA, USA). The RNA samples from different groups were mixed for the subsequent construction of the cDNA libraries.

### 4.2. Library Preparation and SMRT Sequencing

Full-length cDNA was synthesized using the SMARTer cDNA Synthesis Kit (Clontech, Palo Alto, CA, USA) and tissue-specific barcoded oligo dT according to the instructions. The cDNA were fractionated into 3 separate size ranges of 1–2, 2–3 and 3–6 kb in length and validated using BluePippin^®^ Size Selection System (Sage Science, Beverly, MA, USA). The generated cDNA was then re-amplified using PCR. The NEBNext adaptor was ligated to the blunted ends. The products were then digested using the exonuclease/polymerase. The fragment size was selected again to generate proper libraries. The libraries were quantified using the Qubit fluorometer (Life Technologies, Carlsbad, CA, USA). The quality control of the libraries was carried out using the Agilent Bioanalyzer 2100 system. SMRT sequencing was performed on the RSII platform using the C2 sequencing reagents.

### 4.3. Next-Generation Library Preparation and Sequencing

Sequencing libraries were generated using NEBNext^®^Ultra™ RNA Library Prep Kit for Illumina^®^ (NEB, San Diego, CA, USA) following manufacturer’s recommendations and index codes were added to attribute sequences to each sample. Briefly, a total amount of 1 μg RNA per sample was purified using poly-T oligo-attached magnetic beads and then reverse transcribed into cDNA. NEBNext Adaptor was added to the cDNA fragments. Then PCR was performed with Phusion High-Fidelity DNA polymerase, Universal PCR primers and Index (X) Primer. At last, PCR products were purified (AMPure XP system) and library quality was assessed on the Agilent Bioanalyzer 2100 system. After cluster generation, the library preparations were sequenced on an Illumina Hiseq platform and paired-end reads were generated.

### 4.4. Illumina and Pacbio Data Analysis

SMRT-Analysis software package v3.0 (https://github.com/ben-lerch/IsoSeq-3.0/blob/master/README.md) was used for Iso-Seq data analysis. First, reads that have full passes ≥0 and the predicted consensus accuracy >0.75 were classified as ROIs (reads of inserts). Then, reads containing the 5′ and 3′ adapters as well as the poly (A) tail were considered to be FLNC (full-length non-chimeric). Data (raw reads) in fastq format were first processed using in-house perl scripts. Reads containing adapters and poly-N and low-quality reads were removed to generate clean data. Iterative clustering for error correction (ICE) was used to improve consensus accuracy and polished full-length consensus sequences from ICE using RS_IsoSeq (v2.3.0). Short-read Illumina sequencing of the same samples was done to quantify the Iso-Seq non-redundant isoforms. Gene expression levels were estimated by fragments per kilobase of transcript per million fragments mapped. Differential expression analysis of two groups was performed using the DESeq R package (1.10.1) [48]. The resulting *p* values were adjusted using the Benjamini and Hochberg’s approach for controlling the false discovery rate. Transcripts with an adjusted *p*-value < 0.05 found by DESeq were assigned as differentially expressed.

### 4.5. Structure Analysis of Transcripts

Transcripts were screened for putative lncRNAs using four computational approaches, including coding-non-coding index (CNCI), coding potential calculator (CPC), coding potential assessment tool (CPAT), and Pfam database to identify non-protein coding RNA candidates and putative protein-coding RNAs from the unknown transcripts.

Identification of splicing events was performed as following: all sequences were aligned to each other with BLAST16. The alignment results were considered as AS events when sequences were >1000 bp, and there should be two high-scoring segment pairs in the alignment; the AS gap was greater than 100 bp, with at least 100 bp distance to the 3′/5′ end; all alternatively spliced transcripts allowed 5 bp overlap.

Transcripts that were >500 bp were selected for simple sequence repeat (SSR) analysis using the MIcroSAtellite identification tool (MISA; http://pgrc.ipk-gatersleben.de/misa/http://pgrc.ipk-gatersleben.de/misa/).

The coding sequences (CDS) and corresponding amino acid sequences within the transcript sequences were predicted using TransDecoder (https://github.com/TransDecoder/TransDecoder/releases). 

### 4.6. Functional Annotation and Enrichment Analysis

Transcripts were annotated by performing blastx searches against public databases [49], including the non-redundant protein database (Nr) and nucleotide database (Nt) of NCBI, Swiss-Prot, protein family (Pfam), gene ontology (GO), and the Kyoto Encyclopedia of Genes and Genomes (KEGG), with an E-value cutoff of 10^−5^. GO enrichment analysis was performed using topGO with Fisher’s exact test. Pathway enrichment analysis was performed using the KEGG Orthology Based Annotation System (KOBAS) server (version v.3) [50].

### 4.7. Accession Number

RNA-seq data generated in this work have been submitted to the SRA database at NCBI under the accession number PRJNA588136.

### 4.8. Quantitative Real-Time PCR Analysis

A total of 11 pairs of gene-specific primers (Table S5) were designed to produce amplicons for validating the RNA-seq data. Quantitative reverse-transcription PCR (qRT-PCR) was performed on an iQ 5.0 instrument (Bio-Rad, Hercules, CA, USA) using SYBR Green qPCR kits (Roche, Basel, Switzerland) according to the manufacturer’s instructions. Relative gene expression levels were calculated using the 2^−∆∆Ct^ method [51]; expression levels were normalized against the reference gene alpha-tublin. All assays for each gene were performed in triplicates under identical conditions.

## Figures and Tables

**Table 1:** Output statistics.

cDNA Size	Reads of Insert	Number of Filtered Short Reads	Number of Non-Full-Length Reads	Number of Full-Length Reads	Number of Full-Length Non-Chimeric Reads	Average Full-Length Non-Chimeric Read Length	Full-Length Percentage (FL%)
**1–2 K**	201,132	32,182	86,205	82,745	82,235	1475	41.14%
**2–3 K**	217,069	14,850	84,544	117,675	117,094	2392	54.21%
**3–6 K**	88,975	3374	41,772	43,829	43,797	3542	49.26%
**All**	507,176	50,406	212,521	244,249	243,126	2288	48.16%

**Table 2:** GO enrichment analysis of up-regulated transcripts after treatment with different insecticides at different temperatures.

Pesticide	Temperature	GO_ID	GO_Term	p_adj
IM	15	GO:0008238	exopeptidase activity	1.94 × 10^−5^
	25	GO:0008238	exopeptidase activity	8.25 × 10^−5^
		GO:0000062	fatty-acyl-CoA binding	2.42 × 10^−^^2^
	35	GO:0003844	1,4-alpha-glucan branching enzyme activity	3.53 × 10^−4^
		GO:0043169	cation binding	6.95 × 10^−^^3^
		GO:0009982	pseudouridine synthase activity	1.29 × 10^−^^2^
		GO:0016208	AMP binding	2.68 × 10^−^^2^
		GO:0016831	carboxy-lyase activity	3.47 × 10^−^^2^
BCP	15	GO:0008238	exopeptidase activity	8.26 × 10^−^^3^
		GO:0004368	glycerol-3-phosphate dehydrogenase activity	4.42 × 10^−^^2^
	25	GO:0042302	structural constituent of cuticle	0
	35	GO:0016798	hydrolase activity, acting on glycosyl bonds	4.22 × 10^−^^7^
		GO:0004650	polygalacturonase activity	6.40 × 10^−^^6^
		GO:0051287	NAD binding	2.71 × 10^−^^2^
		GO:0004617	phosphoglycerate dehydrogenase activity	3.64 × 10^−^^2^
PHX	15	GO:0004726	non-membrane spanning protein tyrosine phosphatase activity	1.61 × 10^−^^2^
		GO:0004467	long-chain fatty acid-CoA ligase activity	2.42 × 10^−^^2^
	25	GO:0042302	structural constituent of cuticle	4.66 × 10^−^^3^
		GO:0008469	histone-arginine N-methyltransferase activity	3.42 × 10^−^^2^
	35	GO:0000287	magnesium ion binding	1.53 × 10^−4^
		GO:0004450	isocitrate dehydrogenase (NADP+) activity	1.62 × 10^−4^
		GO:0008012	structural constituent of adult chitin-based cuticle	8.06 × 10^−^^3^
		GO:0051287	NAD binding	9.15 × 10^−^^3^

## Supplementary Materials

Supplementary materials can be found at https://www.mdpi.com/1422-0067/21/2/658/s1.

## Abbreviations

IM	Imidacloprid
BCP	Beta-cypermethrin
PHX	Phoxim
DET	Differentially expressed transcript
TC	Temperature coefficient
TOI	Toxicity of insecticides
nTCIs	Negative temperature insecticides
pTCIs	Positive temperature insecticides
neTCIs	No effect temperature insecticides
