# Supplementary material for: Analysis of Differentially Expressed Transcripts in Apolygus lucorum (Meyer-Dür) Exposed to Different Temperature Coefficient Insecticides
Source: Int J Mol Sci. 2020 Jan 19;21(2):658. doi: 10.3390/ijms21020658 (PMC7014463; doi:10.3390/ijms21020658)
Supplement: Supplementary file 1 [file ijms-21-00658-s001.zip › Table S2.docx]

**Table S2.** Statistics table for the number of annotated transcripts

| **#Anno** | **Annotated Number** |
| --- | --- |
| GO | 17,067 |
| KEGG | 17,402 |
| KOG | 24,916 |
| Pfam | 26,771 |
| Swissprot | 22,813 |
| COG | 12,445 |
| eggNOG | 32,825 |
| nr | 34,213 |
| ALL | 34,739 |

Note: Anno is used for the database of functional annotations; Annotated_Number: the number of transcripts for the corresponding database annotation
