# Supplementary material for: Analysis of Differentially Expressed Transcripts in Apolygus lucorum (Meyer-Dür) Exposed to Different Temperature Coefficient Insecticides
Source: Int J Mol Sci. 2020 Jan 19;21(2):658. doi: 10.3390/ijms21020658 (PMC7014463; doi:10.3390/ijms21020658)
Supplement: Supplementary file 1 [file ijms-21-00658-s001.zip › Figure S1.doc]

**Figure S1.** Changes of glutathione, arginine and proline metabolic pathways after imicloprid treatment at 15ºC. Compared to CK15, the enzymes labeled in red box are associated with up-regulated transcripts and the green indicates that of down-regulated transcripts. The number in the box represents the enzyme code.
