# Supplementary material for: Analysis of Differentially Expressed Transcripts in Apolygus lucorum (Meyer-Dür) Exposed to Different Temperature Coefficient Insecticides
Source: Int J Mol Sci. 2020 Jan 19;21(2):658. doi: 10.3390/ijms21020658 (PMC7014463; doi:10.3390/ijms21020658)
Supplement: Supplementary file 1 [file ijms-21-00658-s001.zip › Table S1.doc]

**Table S1. Illumina Hi-Seq output statistics**

| **Sample-ID** | **ReadSum** | **BaseSum** | **GC(%)** | **Q30(%)** |
| --- | --- | --- | --- | --- |
| CK151 | 30534442 | 9147361024 | 47.77 | 93.41 |
| CK152 | 31061473 | 9286197970 | 46.52 | 93.63 |
| CK153 | 23658090 | 7083551870 | 44.61 | 94.47 |
| IM151 | 30545675 | 9135990924 | 46.31 | 93.54 |
| IM152 | 23534695 | 7044763328 | 45.7 | 94.64 |
| IM153 | 30897503 | 9241928560 | 46.94 | 93.78 |
| BCP151 | 26294482 | 7868293482 | 45.23 | 94.76 |
| BCP152 | 25578248 | 7656084468 | 45.1 | 94.13 |
| BCP153 | 23943847 | 7164404976 | 44.85 | 94.73 |
| PHX151 | 24798614 | 7395986308 | 46.97 | 90.86 |
| PHX152 | 21573320 | 6457097994 | 46.83 | 91.17 |
| PHX153 | 24673249 | 7367376362 | 47.27 | 90.84 |
| CK251 | 22828321 | 6833056636 | 45.23 | 94.6 |
| CK252 | 27095017 | 8107943040 | 46.42 | 93.37 |
| CK253 | 31016149 | 9287434516 | 46.18 | 93.41 |
| IM251 | 23472420 | 7016704138 | 46.41 | 90.52 |
| IM252 | 23431067 | 7014328132 | 47.17 | 90.79 |
| IM253 | 23846935 | 7122162974 | 46.87 | 90.84 |
| BCP251 | 24402094 | 7286872478 | 47.32 | 90.91 |
| BCP252 | 25972208 | 7771868002 | 46.94 | 90.69 |
| BCP253 | 27585524 | 8242746588 | 46.74 | 90.65 |
| PHX251 | 27287670 | 8167382698 | 47.32 | 93.81 |
| PHX252 | 21548805 | 6451577626 | 45.09 | 94.32 |
| PHX253 | 27896828 | 8339939384 | 46.85 | 93.01 |
| CK351 | 30469692 | 9116645944 | 45 | 95.1 |
| CK352 | 27640067 | 8262329882 | 47.16 | 93.54 |
| CK353 | 24508657 | 7322363928 | 46.82 | 93.67 |
| IM351 | 22910971 | 6843419504 | 47.67 | 91.45 |
| IM352 | 28368089 | 8490758316 | 46.78 | 91 |
| IM353 | 25078949 | 7493075312 | 47.24 | 91.15 |
| BCP351 | 27039188 | 8076980726 | 47.17 | 91.24 |
| BCP352 | 22404981 | 6699612008 | 47.65 | 91.41 |
| BCP353 | 24804863 | 7409464792 | 47.13 | 91.46 |
| PHX351 | 23958189 | 7157967786 | 46.94 | 91.29 |
| PHX352 | 24684487 | 7374674512 | 47.13 | 91.41 |
| PHX353 | 23385970 | 6996786204 | 46.86 | 91.02 |
| Mean | 25798077 | 7714864789 | 46.56 | 90.52 |
