# Supplementary material for: Analysis of Differentially Expressed Transcripts in Apolygus lucorum (Meyer-Dür) Exposed to Different Temperature Coefficient Insecticides
Source: Int J Mol Sci. 2020 Jan 19;21(2):658. doi: 10.3390/ijms21020658 (PMC7014463; doi:10.3390/ijms21020658)
Supplement: Supplementary file 1 [file ijms-21-00658-s001.zip › Table S3.doc]

**Table S3. Nr annotation of the top20 transcripts with the most isoforms.**

| **ID** | **isoform number** | **nr_annotation** |
| --- | --- | --- |
| PB7838 | 29 | trypsin precursor LhP1 [Lygus hesperus] |
| PB6093 | 18 | trypsin precursor LlGtP1 [Lygus lineolaris] |
| PB3693 | 16 | trypsin precursor LlGtP1 [Lygus lineolaris] |
| PB1320 | 14 | trypsin precursor LlGtP1 [Lygus lineolaris] |
| PB1601 | 14 | trypsin precursor LhP1 [Lygus hesperus] |
| PB9816 | 12 | trypsin precursor LlGtP1 [Lygus lineolaris] |
| PB12540 | 11 | ATP-citrate synthase [Zootermopsis nevadensis] |
| PB7188 | 10 | PREDICTED: SCO-spondin-like, partial [Bombyx mori] |
| PB8689 | 9 | trypsin precursor [Lygus lineolaris] |
| PB3995 | 9 | trypsin precursor LlGtP1 [Lygus lineolaris] |
| PB18346 | 9 | trypsin precursor LlGtP1 [Lygus lineolaris] |
| PB11270 | 9 | trypsin precursor LlGtP1 [Lygus lineolaris] |
| PB1178 | 8 | hypothetical protein TcasGA2_TC010626 [Tribolium castaneum] |
| PB19949 | 8 | ATP-dependent RNA helicase p62 [Culex quinquefasciatus] |
| PB13598 | 8 | protease m1 zinc metalloprotease, partial [Riptortus pedestris] |
| PB8405 | 7 | hypothetical protein TcasGA2_TC010626 [Tribolium castaneum] |
| PB21179 | 7 | trypsin precursor LlGtP1 [Lygus lineolaris] |
| PB18458 | 7 | leucine-rich transmembrane protein [Riptortus pedestris] |
| PB7543 | 6 | PREDICTED: uncharacterized protein LOC100865248 [Apis florea] |
| PB34359 | 6 | cyanoprotein beta subunit precursor [Riptortus clavatus] |
