# Supplementary material for: Analysis of Differentially Expressed Transcripts in Apolygus lucorum (Meyer-Dür) Exposed to Different Temperature Coefficient Insecticides
Source: Int J Mol Sci. 2020 Jan 19;21(2):658. doi: 10.3390/ijms21020658 (PMC7014463; doi:10.3390/ijms21020658)
Supplement: Supplementary file 1 [file ijms-21-00658-s001.zip › Table S4.doc]

**Table S4.** Cluster frequency in KEGG enrichment involving UGT.

| #Kegg_pathway | ko_id | IM35 | BCP15 | BCP25 | BCP35 |
| --- | --- | --- | --- | --- | --- |
| Drug metabolism - cytochrome P450 | ko00982 | 12.40% | 5.12% | 22.58% | 10.53% |
| Metabolism of xenobiotics by cytochrome P450 | ko00980 | 12.40% | 5.12% | 22.58% | 10.53% |
| Drug metabolism - other enzymes | ko00983 | 11.62% | 5.42% | 22.58% | 15.79% |
| Retinol metabolism | ko00830 | 11.63% | 5.12% | 24.19% | 10.53% |
| Ascorbate and aldarate metabolism | ko00053 | 12.40% | 5.42% | 22.58% | 10.53% |
| Starch and sucrose metabolism | ko00500 | 17.83% | 9.04% | 22.58% | 13.16% |
| Porphyrin and chlorophyll metabolism | ko00860 | 12.40% | 5.12% | 22.58% | 10.53% |
| Pentose and glucuronate interconversions | ko00040 | 12.40% | 6.02% | 22.58% | 10.53% |
