# Supplementary material for: Analysis of Differentially Expressed Transcripts in Apolygus lucorum (Meyer-Dür) Exposed to Different Temperature Coefficient Insecticides
Source: Int J Mol Sci. 2020 Jan 19;21(2):658. doi: 10.3390/ijms21020658 (PMC7014463; doi:10.3390/ijms21020658)
Supplement: Supplementary file 1 [file ijms-21-00658-s001.zip › Table S5.doc]

**Table S5. Primers used for qRT-PCR.**

| **Gene name** | **Primer** | **Sequence (5’- 3’)** |
| --- | --- | --- |
| F01.PB34562 **(α-tub)** | Forward primer | GGAACCTACAGACAACTCTTCC |
|  | Reverse primer | ATCTCTTTGCCGATGGTGTAG |
| **PB72** | Forward primer | TTGAGCATGTTGGAGGAATACA |
|  | Reverse primer | TAACCAACCGCTTCGGTAAG |
| **PB1874** | Forward primer | GAACCTGAAGCCTGGTGAA |
|  | Reverse primer | CGAGTAAGGGTTGTAATGTCCTC |
| **PB6512** | Forward primer | CAGTTGCAGCCACATCAAAG |
|  | Reverse primer | CCCTGTACTGCTCCAAAGATAC |
| **PB6447** | Forward primer | CGTGATCCAGGACAAGTACAAG |
|  | Reverse primer | TGAGAGCTAGGAGTTCGATACC |
| **PB13800** | Forward primer | ACTCGCTGAAGACAAGGATAAC |
|  | Reverse primer | CGGACAACTTCTTCCTGTTCT |
| PB3358 | Forward primer | GTTGAACCGATGGCCTACAT |
|  | Reverse primer | GAGTCCTGACAGCTCGTTTATC |
| PB6355 | Forward primer | AACAACGTTTGGGTTGCTTAC |
|  | Reverse primer | CTCCCATGAGTTCAGCGTTAT |
| PB8151 | Forward primer | CGGTATTCATGATGGGACAGTT |
|  | Reverse primer | GTGTTTAGCAGTCACGGAGAG |
| PB7468 | Forward primer | TTGTTCGACTCGGAGGATTG |
|  | Reverse primer | AAGGTTGGCTGGTGTTTCT |
| PB8895 | Forward primer | CTACCCAAATCAGGCCAAAGA |
|  | Reverse primer | CCAATCGACATCAGCTCCATTA |
| PB33113 | Forward primer | GCCAGTTCACCAGAAGATACA |
|  | Reverse primer | GGCAGCTTGAAGAGTGAGAA |
| PB5154 | Forward primer | TCAAGTCCAGGAAAGGAGAAAC |
|  | Reverse primer | CCGTGGATGATAATGCCATACT |
